# The impact of intraoperative radiotherapy on breast cancer: focus on the levels of angiogenic factors

**DOI:** 10.1186/s12957-022-02653-8

**Published:** 2022-06-09

**Authors:** Nahid Nafissi, Maryam Mohammadlou, Mohammad Esmaeil Akbari, Seyed Rabie Mahdavi, Maryam Sheikh, Mohammad Borji, Ebrahim Babaee, Rasoul Baharlou

**Affiliations:** 1 Department of Breast, Rasoul Akram Hospital Clinical Research Development Center (RCRDC), Iran University of Medical Sciences, Tehran, Iran; 2 Cancer Research Center, Semnan University of Medical Sciences, Semnan, Iran; 3 Department of Immunology, School of Medicine, Semnan University of Medical Sciences, Semnan, Iran; 4 Cancer Research Center, Shahid Beheshti University of Medical Sciences, Tehran, Iran; 5 Department of Medical Physics, Iran University of Medical Sciences, Tehran, Iran; 6 Department of Biochemistry, School of Medicine, Shiraz University of Medical Sciences, Shiraz, Iran; 7 Preventive Medicine and Public Health Research Center, Psychosocial Health Research Institute, Department of Community and Family Medicine, School of Medicine, Iran University of Medical Sciences, Tehran, Iran

**Keywords:** IORT, TGF-β, EGF, FGF, VEGF, DLL4, Breast cancer

## Abstract

**Objective:**

Angiogenesis is one of the hallmarks of cancers that is involved in tumor progression. Angiogenic factors induce the formation of new blood vessels and tumor extension, and finally reduce the survival of patients. Intraoperative radiotherapy (IORT), in which radiation is delivered to the tumor bed can kill cells and change tumor microenvironment. Here, we compared the impact of IORT on the levels of angiogenic factors in the blood and surgical wound fluids (SWF) of the breast cancer patients.

**Patients and methods:**

Three hundred sixty patients, who had undergone breast-conserving surgery between 2013 and 2018, were enrolled in IORT and non-IORT groups non-randomly. Blood and drained wound fluid (WF) samples were collected from the patients before and after surgery, followed by quantification of the amounts of TGF-β, EGF, FGF, VEGF, and DLL4 in the patients using ELISA.

**Results:**

Our results were indicative of significant differences between the pre-surgery and post-surgery serum levels of EGF, DLL4, and VEGF. Furthermore, ROC analyses showed that TGF-β and DLL4 can differentiate of the early-stage from late-stage of the disease. Interestingly, the rate of the death and recurrence was reduced in IORT group.

**Conclusions:**

In summary, IORT is a safe and effective treatment that can affect angiogenic factors and improve the overall- and recurrence-free survival of breast cancer patients.

## Introduction

Breast cancer is the most common type of cancer in women [1] and the second most prevalent malignancy in the world [2]. Successful early diagnoses and advanced medical treatments have reduced the mortality rate of the disease by almost 40% over the past 25 years [3]. Angiogenesis is an important phenomenon in a wide variety of normal and cancerous situations. Tumor angiogenesis initiation and development is mainly governed by angiogenic growth factors, such as vascular endothelial growth factor (VEGF), epidermal growth factor (EGF), fibroblast growth factor (FGF) [4], and delta-like 4 (DLL4) [5, 6]. Several *studied* have shown that the levels of angiogenic factors and subsequent formation of new blood vessels can play roles in breast cancer metastasis and relapse-free or overall *survival* [7]. Currently, the standard treatment for breast cancer includes conservative surgery followed by radiation therapy [8, 9]. It has been demonstrated that surgery leads to the elevated survival, proliferation, and migration of the remaining tumor cells in the breast cancer patients [10, 11]. Moreover, during the wound healing process, the immune response or immune cells can promote tumor progression. For example, VEGF and EGF that are secreted by M2 macrophages (tumor-resident macrophages) induce angiogenesis and recruitment of neutrophils which upgrade tumor progression and metastasis [10, 12, 13]. According to a recent study, surgery induces the regrowth of tumor cells thorough the release of cytokines and activation of myeloid cells [14]. It seems that the post-surgical wound fluid collected from the surgical sites stimulates tumor progression through promoting the proliferation and migration of breast cancer cells [14].

Irradiation of the tumor site is the basis of intraoperative radiation therapy (IORT), which is a technique that directly delivers a single high-dose fraction to the tumor bed during surgery [15]. Tumor microenvironment plays a critical role in the risk of breast cancer recurrence. In addition to killing cancer cells, IORT may also act to alter the environment of an irradiated tumor bed [16]. In a study by Belletti et al., IORT (at a dose of 20 Gy) changed the composition of the wound fluid and annihilated its stimulatory effect on the migration, invasion, and proliferation capabilities of the breast cancer cells [17]. It seems that assessing the impact of IORT on the levels of angiogenic factors has an important role in understanding the effectiveness of treatment on the survival of breast cancer patients[18]. In this study, we have investigated the effect of IORT on the levels of angiogenic factors in the blood and surgical wound fluids (SWFs) of patients who underwent breast-conserving surgery (BCS) and subsequent IORT treatment.

## Methods and materials

### Patients and sample collection

This observational study consists of 360 patients, who were diagnosed with breast cancer and recruited in Rasoul Akram and Khatam-al-Anbya hospitals from 2013 to 2018. All of patients were enrolled based on the treatment plan. The non-IORT group consisted of 229 patients in Rasoul Akram Hospital without IORT device that underwent BCS and external beam radiotherapy and the IORT group consisted of 131 patients that were eligible for BCS and subsequently intraoperative radiotherapy to the tumor bed [19, 20]. IORT protocol were based on low and intermediate risk group in the Groupe Européen de Curiethérapie and the European Society for Radiotherapy & Oncology (GEC-ESTRO) recommendations on patient selection for ABPI: patient age ≥ 50 years, histological characteristics: invasive ductal or lobular carcinoma (IDC/ILC), with any histological grade, tumor size ≤ 30 mm, surgical margins < 2 mm, multifocality within 2 cm of the index lesion, any estrogen receptor (ER), and progesterone receptor status [21]. Following wide tumor excision, the eligible patients were prescribed with a single IORT dose using the LIAC HWL (Sordina Electron IORT Technologies, Vicenza, Italy) to the applicator diameter (range 50–70 mm) after putting barrier disk under oncoplastic breast flaps and margin and sentinel node assessment by frozen section. IORT boost and radical dose was performed with a radical single dose of 21 Gy or boost dose of 21 Gy at the surface of the IORT applicator, respectively according to ASTRO and ESTRO protocol. Then, after final pathologic report using, patients who had taken boost dose previously in operating room were prescribed the external radiotherapy (25 sessions) after complete chemotherapy in the department of radiotherapy. At the end, due to importance the glandular flaps to fill the defect of tumor resection, all cases were operated by oncoplastic surgery. The two groups were matched on all clinical variables (Table 1). Five milliliters peripheral venous blood samples were collected before surgery form patients. Twenty-four to 36 h after surgery, 5 mL peripheral blood and drainage WF samples were collected from both groups and after centrifugation, they were stored at – 80 °C. The protocol for the present study was approved by the Ethics Committee of Semnan University of Medical Sciences (IR.SEMUMS.REC.1398.58). Informed consent was obtained from all subjects who participated in this study. The characteristics of patients have been summarized in Table 1.

**Table 1:** Demographic, clinical, and laboratory data of breast cancer patients with IORT and non-IORT

Variables	Non-IORT (***n*** = 229)	IORT (***n*** = 131)	***p***
**Age**	50 ± 63	51 ± 31	NS
**Tumor side (%)**			NS
**Right**	116 (78.5)	62 (47.3)	
**Left**	113 (21.5)	69 (52.7)	
**Family history (%)**			0.01
**Negative**	176 (76.8)	85 (65.5)	
**Positive**	53 (23.2)	46 (34.5)	
**Histology (%)**			NS
**IDC**	176 (76.8)	99 (75.5)	
**ILC**	35 (15.3)	20 (15.3)	
**DCIS**	14 (6.2)	11 (8.3)	
**Other types**	4 (1.7)	1 (0.8)	
**ER (%)**			0.01
**Negative**	86 (37.5)	33 (25.2)	
**Positive**	143 (62.5)	98 (74.8)	
**PR (%)**			NS
**Negative**	89 (38.9)	43 (32.8)	
**Positive**	140 (61.1)	88 (67.2)	
**HER-2 (%)**			NS
**Negative**	141 (67.3)	91 (71.7)	
**Positive**	66 (32.7)	36 (28.3)	
**Tumor grade (%)**			NS
**I (well differentiation)**	26 (11.4)	18 (13.7)	
**II (mod differentiation)**	119 (51.9)	80 (61.1)	
**III (poor differentiation)**	84 (36.7)	33 (25.2)	
**Vascular invasion (%)**			NS
**Negative**	147 (64.2)	95 (72.5)	
**Positive**	82 (35.8)	36 (27.5)	
**Perineural invasion (%)**			NS
**Negative**	198 (86.5)	112 (85.5)	
**Positive**	31 (13.5)	19 (14.5)	
**Calcification (%)**			NS
**Negative**	185 (83.7)	114 (87.0)	
**Positive**	36 (16.3)	17 (13.0)	
**Necrosis (%)**			NS
**Negative**	170 (76.4)	95 (72.5)	
**Positive**	51 (23.6)	36 (27.5)	
**Pathological T stage (%)**			NS
**T1 (≤ 2 cm)**	100 (43.6)	64 (48.8)	
**T2 (> 2 cm, ≤ 5 cm)**	120 (52.4)	67 (51.2)	
**T3 (> 5 cm)**	9 (3.9)	0 (0)	
**Pathological N stage (%)**			NS
**N0**	122 (53.3)	83 (63.4)	
**N1 (1–3)**	59 (25.7)	29 (22.1)	
**N2 (4–9)**	23 (10.1)	12 (9.2)	
**N3 ≥ 10**	25 (10.9)	7 (5.3)	
**TNM stage (%)**			NS
**0**	14 (6.1)	11 (8.5)	
**I**	52 (22.7)	41 (31.8)	
**II**	105 (45.8)	57 (44.2)	
**III**	56 (24.4)	20 (15.5)	
**IV**	2 (1.0)	0 (0)	

Values are presented as mean ± standard deviation or number (%). *IORT* intraoperative radiation therapy, *DCIS* ductal carcinoma in situ, *IDC* invasive ductal carcinoma, *ILC* invasive lobular carcinoma, *ER* estrogen receptor, *PR* progesterone receptor, *HER2* human epidermal growth factor receptor 2, *TNM* tumor, node, and metastasis

### Enzyme-linked immunosorbent assay (ELISA)

The amounts of TGF-β, EGF, FGF, VEGF, and DLL4 in the patients’ sera were measured using ELISA-kits which were purchased from eBiosciences (USA), Thermo Fisher (USA), and Fine Biotech (China), respectively. Briefly, standards were reconstituted to generate stock concentrations of 500, 5000, 10,000, 10,000, and 5000 pg/mL for TGF-β, EGF, FGF, VEGF, and DLL4, respectively. The detection sensitivity for TGF-β, EGF, FGF, VEGF, and DLL4 was 8, 1, 15.6, 5, and 46.9 pg/mL, respectively. Briefly, the diluted Capture Antibodies were added to 96-well microtiter plates for overnight. Then Standards or samples were added, and incubated for overnight at 4 °C. After washing, the detection antibody was added and incubated for 2 h at room temperature. After a series of washes, Streptavidin-HRP and then, 50 μL of Stop Solution were added. Finally, the plate was read using a micro-plate reader set to 450 nm. Data were expressed in pg/mL.

### Statistical analysis

All statistical calculations were conducted using the Prism 8.0.2 (GraphPad v7, USA) and SPSS (SPSS, v17, USA) software. After assessment of data normality, the serum levels of TGF-β, EGF, FGF, DLL4, and VEGF in the peripheral blood and drainage WF were evaluated compared to the corresponding values from control samples using independent *t* test or Mann-Whitney *U* test, paired *t* test, or Wilcoxon matched-pairs rank test. The diagnostic accuracies of disease stage, LN involvement and tumor size were evaluated using the receiver operating characteristic (ROC) analysis and the areas under the ROC curves (AUCs) were compared for each serum variable. We presented the categorical variables as frequencies and proportions and the results were compared using the chi-squared test. The overall survival and recurrence-free survival (relapse-free survival) of the different groups of patients from surgery to last follow-up were estimated by the Kaplan–Meier survival curve and evaluated by the log-rank tests. Results were expressed as mean ± SD and *p* < 0.05 was regarded as significant in all statistical analyses.

## Results

### Evaluation of clinical parameters

Among 360 patients under investigation, 229 patients were in the non-IORT group with the mean age of 50 ± 13 years and 131 patients were in the IORT group with the mean age of 51 ± 10 years. Furthermore, 45 (18.8 %) patients in the non-IORT group and 41 (34.5%) in the IORT group had family history of BC, while 103 (51.8%) patients in the non-IORT and 74 (61.7%) ones in the IORT group had grade II tumors (moderate-differentiation). The most predominant tumor type in the studied patients was ductal, which was observed in 171 patients (77.4%) in the non-IORT group and 98 patients (75.4%) in the IORT group. Demographic, clinical, and laboratory data of the breast cancer patients in the two studied groups have been shown in Table 1.

### The impact of IORT on levels of angiogenic factors in the sera of breast cancer patients

**Fig. 1:**
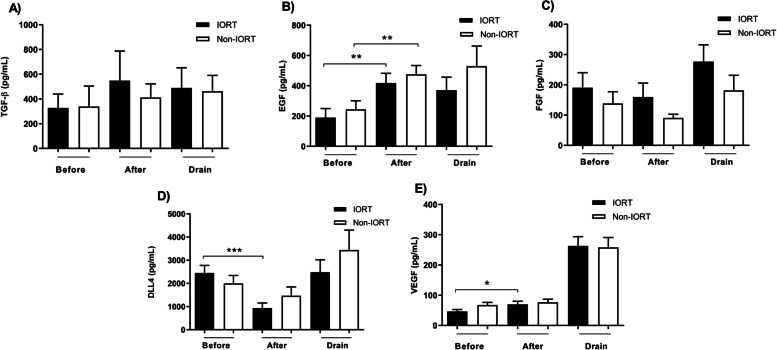
The level of TGF-β, EGF, FGF, DLL4, and VEGF in the peripheral blood and WF of breast cancer patients with and without IORT. Significant difference was found in the serum level of EGF (**B**), DLL4 (**D**), and VEGF (**E**) between before and after IORT. Results were analyzed with non-parametric Wilcoxon matched-pairs rank test and two-tailed Mann-Whitney *U* test. Values are the mean ± SEM; * = *p* < 0.05; ** = *p* < 0.01

We evaluated the levels of TGF-β, EGF, FGF, DLL4, and VEGF in the peripheral blood and WF of breast cancer patients in the IORT and non-IORT groups. According to the results, significant differences were found in the serum levels of EGF, DLL4, and VEGF measured before and after IORT (Fig. 1). We found that in both IORT and non-IORT groups, EGF concentration was increased after surgery (Fig. 1B). However, DLL4 was decreased after intervention in the IORT group (Fig. 1D). Similar to the case of EGF, VEGF level was increased in the IORT group after surgery (Fig. 1E).

### The differentiation of stages of disease using angiogenic factors

**Fig. 2:**
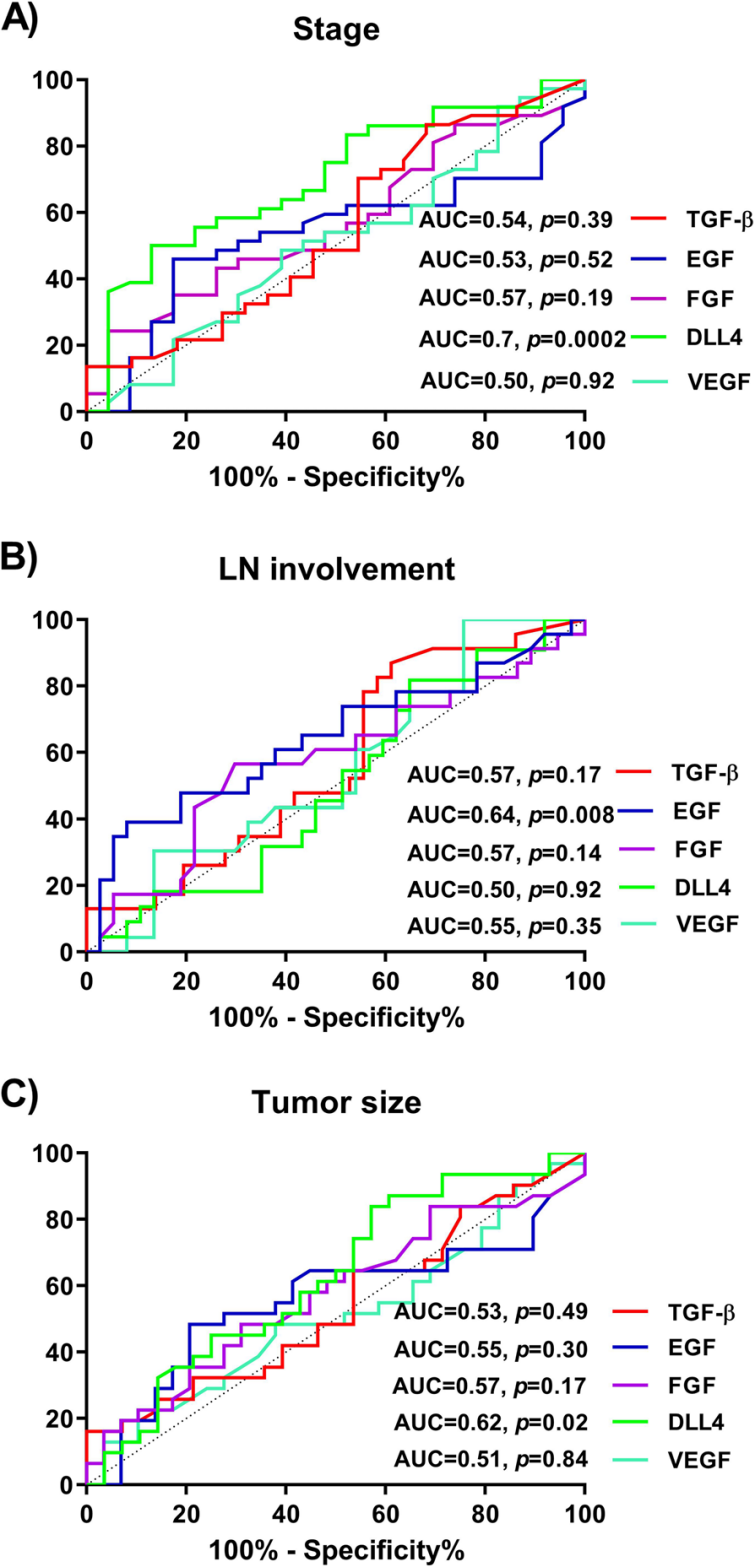
Receiver operating characteristics (ROC) curve analyses of levels of TGF-β, EGF, FGF, DLL4 and VEGF in the stage, LN involvement and tumor size groups. (**A**) As diagnostic biomarkers differentiating early from late stage. (**B**) Diagnostic biomarkers differentiating LN free from > 1 LN involvement. (**C**) Diagnostic biomarkers differentiating tumor size ≤ 2 from > 2 cm. AUC: area under the curve; LN: lymph node

As can be seen from the Fig. 2, using ROC analysis, DLL4 and EGF can be used to differentiate patients with late stages, LN involvement and larger tumor size from early stage, LN free and smaller tumor size, respectively. DLL4 with 70% specificity and sensitivity (AUC = 0.7, *p* = 0.0002) (Fig. 2A) could be employed to differentiate early from late stages to predict the stage of disease. On the other hand, EGF with 64% specificity and sensitivity (AUC = 0.64, *p* = 0.008) (Fig. 2B) could be utilized to differentiate LN free from > 1 LN involvement for predicting LN involvement. In addition, tumor size could be predicted with 62% specificity and sensitivity using DLL4 (AUC = 0.62, *p* = 0.02) (Fig. 2C).

### The impact of IORT on survival and recurrence rate in breast cancer patients

**Fig. 3:**
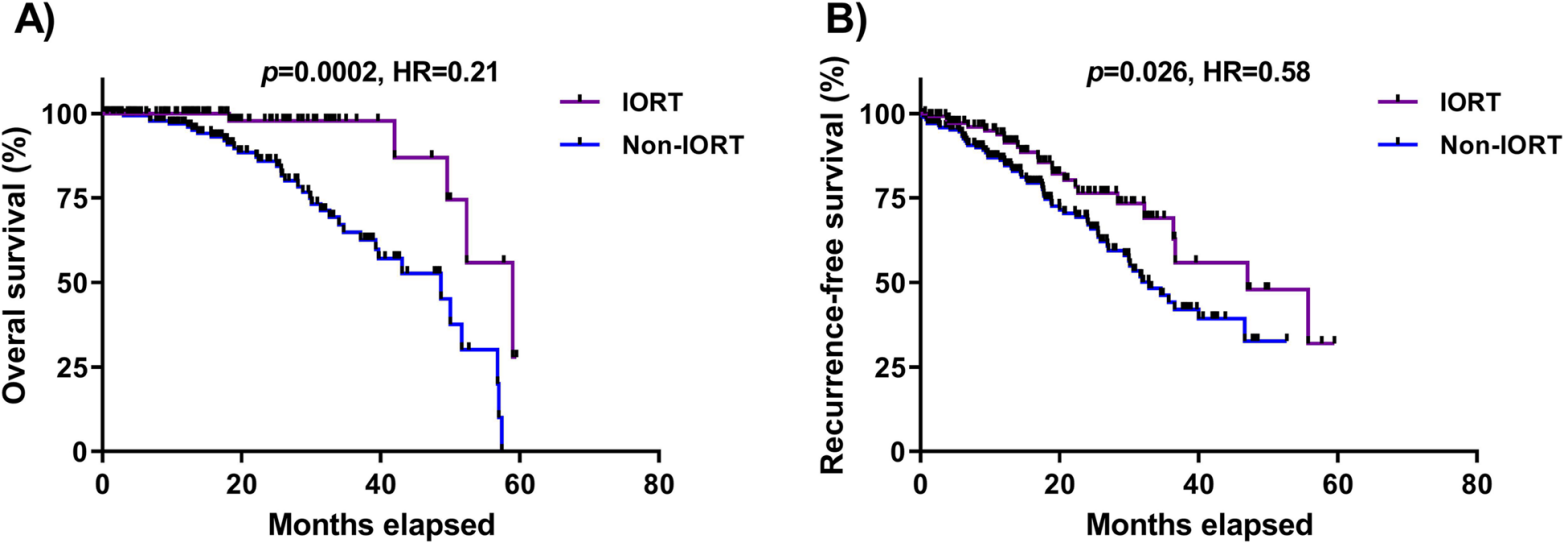
Kaplan–Meier analysis of survival rate (**A**) and recurrence rate (**B**) in IORT and non-IORT groups. Significant differences were determined by log-rank (Mantel–Cox) analysis for (**A**) overall survival (IORT median survival 59.00 and non-IORT median survival 48.70) and (**B**) recurrence-free survival (IORT median survival 47.03 and non-IORT median survival 32.87). IORT: intraoperative radiotherapy

To determine whether IORT could contribute to the improved overall and recurrence-free survival, we plotted Kaplan–Meier survival curves for the patients. As observed in Fig. 3, IORT reduced the risk of the death (HR = 0.21, *p* = 0.0002) (Fig. 3A) and recurrence rate (HR = 0.58, *p* = 0.026) and (Fig. 3B) in comparison to the non-IORT group.

## Discussion

Tumor angiogenesis is a highly complex process that involves accurate communication of tumor cells with their host organs or tissues, which is controlled by the interplay of a wide range of factors [22]. Angiogenic factors encourage the formation and development of blood vessels by cancer cells to expand tumors [23]. Several studies have shown that angiogenic factors have a significant role in tumor growth and expansion. According to the outcomes of immunohistochemical analyses, the members of VEGF family and their receptors are expressed in almost half of human cancers. Moreover, a significant association between the expression of VEGF and prognosis has been described in colorectal, breast, lung, head and neck squamous cell carcinomas, Kaposi sarcoma, and malignant mesothelioma. These researches have also demonstrated that the levels of angiogenic factors in a tissue indicate the aggressiveness of tumor cells, and thus have predictive value in recognizing patients with poor prognosis who are at high risk [24].

Several studies have shown that overexpression of angiogenic factors such as PD-ECGF, bFGF, TGF-β, angiogenin, and COX-2 in different cancers are correlated with the advanced tumor stage and decrease patient survival [25]. Aside from the VEGF family, FGFs are also known as a family of potent angiogenic motivators associated with the risk of breast cancer [26]. While FGF-1 is known as an acidic polypeptide, FGF-2 is a bFGF polypeptide and plays a pivotal role in the stimulated proliferation and differentiation of endothelial cells. In addition, a significant association between high serum or urine levels of bFGF and progressive disease in patients with different types of cancers has been reported [26, 27].

TGF-β is secreted by both of normal and cancerous cells. Depending on the stage of breast cancer development and progression, it can act as either a pro- or anti-oncogenic protein. TGF-β is a highly oncogenic factor in the late stage, aggressive and metastatic breast cancers [28]. According to recent studies, the high expression of angiogenesis-related proteins is associated with adverse clinicopathological parameters in the early-stage breast cancer patients [29].

Radiation can cause damage to the microenvironment of both cancerous and normal cells (like endothelial cells). There are conflicting reports on the consequences of radiation. Some studies indicate that radiation may enhance tumor invasiveness and metastasis. These observations may be explained by the fact that cancer cells are destroyed or damaged by radiation, thereby secreting a variety of soluble factors that promote angiogenesis and improve migration and invasion of cancer cells [30, 31]. Destruction of epithelial cells by radiation depends on the beam dose. It has been generally reported that higher doses in the range of 2–15 Gy have an anti-angiogenic effect, while lower doses about 0.5–0.8 Gy appear to be pro-angiogenic [32, 33]. However, it can be more complicated than it seems. For instance, it has been reported that a single high dose (20 Gy) of radiation to the mammary gland decreases the local vessel density in a mouse model of breast cancer relapse, after injection of tumor cells [34]. Radiation has been reported to alter the expression of cytokines in the wound fluid [17]. Furthermore, IORT changes the expression of miRNA223, thereby reducing EGF expression and EGF receptor activation, a cascade that normally inhibits the growth of breast cancer cells and decreases the risk of local tumor recurrence in mice models [35].

In the present research, the concentration of EGF in both IORT and non-IORT groups was increased after surgery. Our results also indicated that IORT decreases the DLL4 level. In addition to VEGF, EGF level was also increased after IORT intervention. DLL4 is a critical factor in vascular maturation and tumor angiogenesis and plays a key role in VEGF signaling [6]. A recent study has shown that VEGF secretion by tumor cells is essential for tumor development in the early-stage of breast tumors [36].

An attractive finding obtained from the ROC analysis was that DLL4 and EGF levels can be used to differentiate the late stages of disease from early stages, LN involvement from free LN, and high tumor size from low tumor size. According to the results of previous studies, the serum level of TGF-β is an early marker for predicting fibrosis after surgery and before radiotherapy. The serum levels of TGF-β in patients who had undergone IORT after surgery were significantly higher than those of the patients that had only undergone breast-conserving cancer surgery, suggesting that this alteration in the TGF-β level was the outcome of IORT [37].

Keegan et al. have demonstrated that young patients with breast cancer are associated with more advanced stages, such as higher T and N stages [38]. Another interesting finding was the effect of IORT on the recurrence-free survival. We demonstrated that treatment with IORT reduces the risk of the death and recurrence rate in comparison to the non-IORT group. Furthermore, several randomized trials have demonstrated excellent early tumor control, survival, and cosmetic outcomes following IORT in the breast cancer patients [39]. Vaidya et al. have performed a prospective randomized study on the IORT treatment versus the whole-breast radiotherapy based on a four-year dataset. They demonstrated a local recurrence rate of 1.2% in the IORT group versus 0.95% in the external beam radiotherapy group [19]. In another research, they have reported a 5-year risk for local recurrence in the conserved breast equal to 3.3% for targeted intraoperative radiotherapy (TARGIT) versus a value of 1.3% for the adjuvant whole-breast external beam radiotherapy (EBRT). However, the mortality due to breast cancer in the TARGIT vs EBRT groups were the same [40].

In our study, the mean sample collection period after surgery was 24 to 36 hours after surgery, while if it had been postponed till 72 to 96 h, it might have been led to more accurate results on the effect of IORT on the angiogenic factors. In this way microenvironment had more time to influence on angiogenic factors following IORT. Another limitation of the present study was the possibility effect of the difference in providing services of the Rasoul Akram and Khatam-al-Anbya hospitals on the amount of observed factors. First one is government hospital without IORT instrument that admits more patients than Khatam-al-Anbya hospital that may influence observed outcomes. However, all patients have been following up for more than 5 years now that it seems IORT could offer a potential survival advantage that help to reduce rate of recurrence and death.

## Conclusion

In general, current study, in addition to the well-known tumoricidal effects of IORT, provides a biological basis for intervention that demonstrates the effects of this treatment on reducing tumor recurrence through alterations in the tumor microenvironment and angiogenic factors. IORT can be regarded as an innovative approach for the delivery of efficient radiation to the tumor bed and improve the survival of breast cancer patients with less toxic effects. These findings may also help us in early detection of end-stage of disease, based on the levels of angiogenic factors in patients with breast cancer.

## Data Availability

All data generated or analyzed during this study are included in this article. Further enquiries can be directed to the corresponding author. A preprint version of this article is available on research square [18].
